# GLI2 Regulates TGF-β1 in Human CD4^+^ T Cells: Implications in Cancer and HIV Pathogenesis

**DOI:** 10.1371/journal.pone.0040874

**Published:** 2012-07-31

**Authors:** Robert L. Furler, Christel H. Uittenbogaart

**Affiliations:** 1 Department of Microbiology, Immunology, and Molecular Genetics, David E. Geffen School of Medicine, University of California Los Angeles, Los Angeles, California, United States of America; 2 Department of Pediatrics, David E. Geffen School of Medicine, University of California Los Angeles, Los Angeles, California, United States of America; 3 UCLA AIDS Institute, University of California Los Angeles, Los Angeles, California, United States of America; 4 Jonsson Comprehensive Cancer Center, David E. Geffen School of Medicine, University of California Los Angeles, Los Angeles, California, United States of America; New York University, United States of America

## Abstract

Elevated levels of the immunoregulatory cytokine TGF-β1 in cancer and HIV infection have been linked to the suppression of protective immune responses. The transcriptional regulation of TGF-β1 is complex and still not completely understood. We report here for the first time that the transcription factor GLI2 regulates the expression of TGF-β1 in human CD4^+^ T cells. *In silico* screening revealed five novel putative GLI binding sites in the human *TGF-β1* promoter. At least two of these sites within the human *TGF-β1* promoter are regulated by the GLI2 activator as knockdown of *GLI2* in regulatory CD4^+^CD25^hi^ T cells, high producers of TGF-β1, significantly decreased *TGF-β1* transcription. Additionally, naïve CD4^+^ T cells, low producers of TGF-β1, increased their basal level of *TGF-β1* mRNA following lentiviral infection with GLI2. The transcriptional regulation of *TGF-β1* by GLI2 is a new extension to Sonic Hedgehog (SHH) and *TGF-β1* cross-regulation and may provide insight into the detrimental elevation of TGF-β1 leading to pathogenesis in cancer and HIV infection.

## Introduction

Transforming Growth Factor-β1 (TGF-β1) is a pleiotropic cytokine that regulates the immune response in many diseases including cancer and HIV infection and plays an important role in preventing pathogenesis[1]–[5]. It is produced by several cell types including CD4^+^CD25^hi^ regulatory T cells, macrophages, as well as by fibroblasts. TGF-β1 decreases proliferation of T and B lymphocytes, effector functions of many cells of the immune system and induces peripheral regulatory T cells [6]. Although TGF-β1 is critical throughout mammalian development and to prevent aberrant activation of the host’s immune system, elevated expression of TGF-β1 can dampen protective immune responses to tumors and infections.

Aberrant high production of TGF-β1 by malignant cells was shown to increase metastasis as well as to prevent a protective host immune response to tumors [7]. In chronic HIV infection, TGF-β1 is elevated in several compartments including lymphoid tissues, blood, and cerebrospinal fluid[8]–[10]. Increased levels of TGF-β1 can induce the development of induced (i)Treg observed in chronic HIV infection [11], [12], and may contribute to dysfunction of the immune system during late-stage disease. The HIV-1 Tat protein induces TGF-β1 in human leukocytes [13]–[15], chondrocytes [16], and also in murine CD2^+^ T cells of a Tat-transgenic mouse model [17]. The mechanisms of tumor-induced and Tat-mediated TGF-β1 induction are unknown. Cancer and AIDS are only two of the many diseases in which TGF-β1 plays a role in pathogenesis.

Expression of active TGF-β1 is tightly regulated at the transcriptional, translational, and post-translational levels. Although TGF-β1 expression has been a focus of intense investigation, we are still far from understanding this cytokine’s complex regulation. *In silico* analysis of the TGF-β1 promoter region indicates several putative binding sites of known transcription factors. Some of these transcription factors, like Sp-1 and Zf9, have previously been found to mediate *TGF-β1* transcription, but these factors alone are not sufficient to induce mRNA expression [18], [19]. Therefore we investigated which other transcription factors induce TGF-β1 mRNA.

We found six putative binding sites (five previously unreported) for the human GLI transcription factors by *in silico* analysis of the human *TGF-β1* promoter. The GLI2 transcription factor is one of the main downstream effectors of the Sonic Hedgehog (SHH) pathway, which was shown to play a role in the function of the adaptive immune system [20], [21]. The SHH pathway modulates activation [22], proliferation [23], [24], and cytokine regulation [25] in CD4^+^ T cells. Since several interactions between the TGF-β1 and SHH pathways exist (Table 1), we focused on regulation of TGF-β1 by SHH pathway family members, i.e. GLI proteins, in the immune system.

**Table 1:** The TGF-β and SHH Pathways Exhibit Extensive Cross-Regulation.

ASSOCIATION	REFERENCE
Constitutive SHH pathway activation in transgenic mouse have elevated TGF-β1 transcription.	(Eichenmuller *et al.*, 2007)
GLI3 binds to SMAD proteins.	(Liu *et al.*, 1998)
BMP-2, 4, & 7 (TGFβ family members) all have GLI-responsive elements in their promoters.	(Miyashita *et al.*, 2008; Suguira T., 1999; Kawai, S. and T. Sugiura, 2001)
TGF-β2 promoter contains a SHH-responsive element.	(Lin *et al.,* 2006)
TGF-β1 and SMAD3 upregulate GLI2 and GLI1	(Dennler *et al.*, 2007; Dennler *et al.,* 2009)

We provide evidence that at least two of the six putative GLI-binding sites are required for GLI2-mediated transcriptional regulation of the human *TGF-β1* promoter. At least one of these two sites must be functional to observe TGF-β1 regulation by GLI2. Furthermore, by knocking down *GLI2* in human CD4^+^CD25^hi^ Treg, we show that GLI2 is necessary for TGF-β1 expression. The transcriptional regulation of *TGF-β1* by GLI2 has not yet been described and increases our understanding of the complexities of *TGF-β1* regulation.

## Materials and Methods

### Ethics Statement

The use of anonymous peripheral blood mononuclear cells to obtain T cells and the epithelial 293T cell line was reviewed by the UCLA Institutional Review Board (IRB), which concluded that this activity did not involve human subjects, and therefore did not require IRB review or certification.

### In Silico Screening

Screening for putative GLI binding sites in the human *TGF-β1* promoters was done by *in silico* analysis using the software program MatInspector (http://www.genomatix.de). A range of twenty kilobases surrounding the first human *TGF-β1* promoter were used to screen for putative transcription factor binding sites. All six putative GLI binding sites within the first kilobase of *TGF-β1* promoter #1 (five potential sites) or promoter #2 (one potential site) had a matrix similarity score equal to or greater than 0.8. Binding sites found within the first proximal promoter region are novel and are the focus of this study.

### Luciferase & Promoter Studies

The human *TGF-β1* promoter-luciferase reporter constructs, phTG5 and phTG1, were kindly provided by S.J. Kim [18]. The wild-type phTG5 construct was mutated at the putatitve GLI binding sites (site A, B, or A and B) using site-directed mutagenesis (SITE A: 5′-CAGCCCCCCCATGCC-3′ to 5′-CAGCaaCaaaATGC-3′, SITE B: 5′-GAGCCCGCCCACGCGA-3′ to 5′-GAGCaaGaaaACGCGA-3′, mutated bases in lower case). The wild-type phTG1 construct was mutated at putative GLI binding Site E using site-directed mutagenesis (SITE E: 5′-CCCACCACCCACGAA-3′ to 5′CCaAaaAaaaACGAA-3′, mutated bases in lower case). The wild-type promoter, the mutated promoters, or a pGL3 empty-vector control were individually co-transfected with the constitutive activator vector pGLI2ΔN (kindly provided by E. Roessler [26]) or the control pcDNA3 with Lipofectamine 2000 using standard procedures into 293T cells, a human epithelial cell line containing Adeno and SV-40 viral DNA sequences, obtained from the American Type Culture Collection (ATCC). The pGLI2ΔN plasmid contains a constitutively active GLI2 gene due to an amino-terminal truncation. The amino terminal domain of the human GLI2 gene has a repressive function, and it’s removal has been shown to increase transcription of downstream target genes such as GLI1 [26]. The 293T cell line was chosen because of its transfection efficiency and low levels of GLI1 expression, which is our readout for GLI2 activation. Luciferase expression was measured at 48 hours post-transfection.

### Cell Isolation, Culture, & Stimulation

Naïve CD4^+^ T cells, expressing CD4, CD31, CD45RA, and CD62L, were purified and isolated from human peripheral blood mononuclear cells (PBMC) using negative selection. CD4^+^CD25^hi^ regulatory T cells were enriched from human blood using RosetteSep and Robosep kits from Stem Cell Technologies. Primary cells were cultured in serum-free medium consisting of Iscove’s Modified Dulbecco’s Medium supplemented with delipidated BSA (Sigma-Aldrich) at 1100 µg/mL, human transferrin (Sigma-Aldrich) at 85.5 µg/mL, 2 mM glutamine, and penicillin/streptomycin at 25 U/25 µg/mL. All stimulation conditions of primary T cells were done with αCD3/αCD28-coated M-450 beads from Invitrogen. Cell surface and intracellular immunophenotypes were validated by flow cytometry as described previously [27]. For detection of intracellular FoxP3, cells were first stained for cell surface markers, fixed and permeabilized with eBioscience recommended buffers following manufacturer instructions, then incubated with FITC or eFluor 450 conjugated monoclonal antibodies against FoxP3 (eBioscience, clone PCH101).

### siRNA Knockdown

Knockdown of *GLI2* in primary human regulatory T cells was done using ACCELL siRNA designed and validated by ThermoFisher/Dharmacon. A non-targeting siRNA was used as a negative control. A positive control siRNA for GAPDH was used to verify knockdown. Knockdown of GLI2 was done using a commercially available and validated siRNA from Dharmacon (A-006468-14 - Target sequence - 5' GGUUUGAAUCUGAAUGCUA 3'). A separate independent negative control using a scrambled *GLI2* siRNA sequence (5'- AAGGUAUGCGCUUAAUUU-3') was used to address off-target effects. *GLI2* knockdown was verified by assessing *GLI1* mRNA expression following stimulation. GLI1 has been previously shown to be transciptionally regulated by GLI2 and is the positive control for *GLI2* knockdown.

Purified CD4^+^CD25^hi^FoxP3^+^regulatory T cells were incubated with the siRNA and serum-free ACCELL medium (Dharmacon) for 72 hours prior to stimulation with αCD3/αCD28 coated beads. Twenty-four hours post-stimulation, cells were collected to measure *TGF-β1* mRNA levels by RT-PCR. TGF-β1 levels were normalized to *18S rRNA* and compared to unstimulated cells.

### RT-PCR

Taqman Gene Expression primer/probe sets for *TGF-β1, GLI1*, 18S rRNA, and *GAPDH* from Applied Biosystems Inc. were used to measure transcripts levels in various conditions. The expression of *TGF-β1, GLI1*, and *GAPDH* were normalized to 18S rRNA levels and relative expression between conditions was calculated to assess upregulation/downregulation of transcription.

### Lentiviral Infections

The *GLI2ΔN* transgene was placed into a third-generation HIV-1 vector at the UCLA Vector Core. The majority of the HIV viral coding sequence was removed and replaced by the transgene. The LTRs were modified to be "self-inactivating,” and the transcription of the vector genome in the packaging cells was driven by a non-HIV promoter. Virus was made using 293T cells that were co-transfected with the vector plasmid and three other plasmids encoding A) the viral enzymes and structural proteins, except Env, B) Rev, which is involved in HIV RNA transport, and C) VSV glycoprotein, which is a pan-tropic envelope that allows virus entry into almost any cell type. Naïve CD4^+^ T cells were infected with 100 ng p24 per million cells in serum-free medium for 96 hours. RNA was isolated for determination of changes in gene expression using RT-PCR. A GFP-containing lentivirus was used as a negative control and also to measure infection efficiency.

### Chromatin Immunoprecipitation (ChIP)

**Figure 1:**
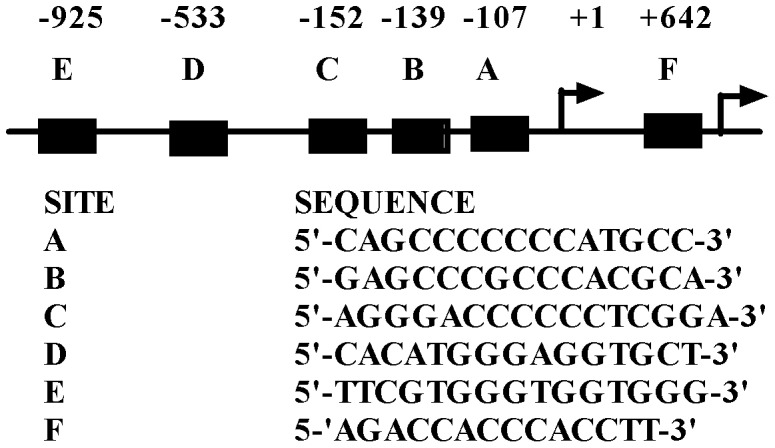
Five Novel Putative GLI Binding Sites Lie Within the First Human TGF-β1 Promoter. There are two reported promoters in the human *TGF-β1* gene. *In silico* analysis of the human *TGF-β1* proximal promoter regions using the Genomatix software program revealed five previously unidentified putative GLI-binding sites (A through E) in a highly conserved region of the human genome. Site F in the second promoter has previously been identified and studied [28]. Sites A and B (which overlaps with C), all lie within the luciferase reporter construct phTG5 used in the promoter mutagenesis studies. Site E is present in the phTG1 construct. Approximate base pair positions of potential GLI binding sites are shown relative to the two reported transcription start sites for the human *TGF-β1* gene.

ChIP analysis was done to determine whether GLI2 binds to the *TGF-β1* promoter. Regulatory CD4^+^ CD25^hi^ T cells were negatively isolated as described above. The cells were stimulated with αCD3/αCD28-coated beads overnight. The GLI2 ExactaChIP kit (R&D Systems) was used for chromatin immunoprecipitation. Cells were fixed, lysed, and sonicated prior to incubation with either αGLI2-coated or control αIgG-coated agarose beads. PCR was done following ChIP to assess GLI binding to the *TGF-β1* promoter. Putative GLI2 binding site ‘E’ (Figure 1) was detected using designed primers (5′-CTGGGGTCAGCTCTGACAGT-3′ and 5′-CAGTTGGCGAGAACAGTTGG-3′; 290 bp fragment). The *Bcl2* promoter region was detected as a positive GLI-binding control using primers from the ExactaChIP kit. A 238 bp region of the *IL-10* promoter which contained no putative GLI2 binding site was measured as a negative control (5′-TGATTTCCTGGGGAGAACAG-3′ and 5′-CCCACCCCCTCATTTTTACT-3′).

### Co-Immunoprecipitation

Co-immunopreciptation was performed to assess the binding of human GLI2 to HIV-1 Tat. Dual transfections were done in 293T cells using three different sets of plasmids: (GLI2-6xMYC + HIV-1 Tat-FLAG), (GLI2-6xMYC + PCDNA3 control), (HIV-1 Tat-FLAG + PCDNA3 control). Forty-eight hours post-transfection, the cells were lysed and the released proteins were collected for co-IP (Pierce Co-IP Kit from Thermo Scientific). The agarose resin was coated with one of three antibodies: Sigma F1804 anti-FLAG M2 antibody (to bind Tat to bead), CST 71D10 anti-MYC antibody (to bind GLI2 to bead), or an isotype control antibody. Following co-IP, the elutions were run on an SDS-PAGE gel followed by Western Blotting.

### Statistics

All analyses were conducted by the UCLA AIDS Institute Biostatistics Core or with GraphPad Prism 5 Software. Variables were expressed as means with standard error of the mean. ANOVA and two-tailed Wilcoxon Rank sum test, paired when appropriate, were used to analyze differences between populations. A p value lower or equal to 0.05 was considered significant.

## Results

### Six Putative GLI Binding Sites Lie Within the Human *TGF-β1* Promoters

Through *in silico* analysis using MatInspector (http://www.genomatix.de) we found six putative GLI binding sites within the two reported human *TGF-β1* promoters, including a previously reported site within the second promoter [28] in addition to five sites which were previously unidentified. Both Genomatix software and UCSC genome (www.genome.ucsc.edu) programs indicate a high degree of sequence conservation surrounding the *TGF-β1* promoter region in mammals which suggests the functional relevance of the human *TGF-β1* promoters. The consensus binding sequence for the GLI transcription factors has been identified as 5′-TGGGTGGTC-3′ [29], [30]. The six putative GLI binding sites reported in this study have a high-degree of matrix similarity^43^ and are listed in Figure 1. The abundance of possible GLI binding sites within the first thousand base pairs of the transcription start site of the *TGF-β1* promoter suggests that this proximal promoter region may be regulated by the GLI transcription factors.

### GLI2 Activates the First Human *TGF-β1* Promoter Through At Least Two of the Putative GLI-binding Sites

The putative GLI binding site in the second TGFβ-1 promoter has been reported to be non-responsive to SHH stimulation by Eichenmuller *et al.* [28]. However, the five putative sites in the first promoter have not previously been reported to be regulated by GLI proteins. We used a reporter construct containing the first 500 base pairs of the human TGF-β1 promoter which was placed upstream of the firefly luciferase gene by Kim *et al.* (phTG5) [18]. This expression vector was co-transfected into 293T cells along with a constitutive GLI activator construct pGLI2ΔN [26] or a pcDNA3 plasmid control to assess GLI2-mediated regulation of the promoter of the human TGF-β1 gene.

**Figure 2:**
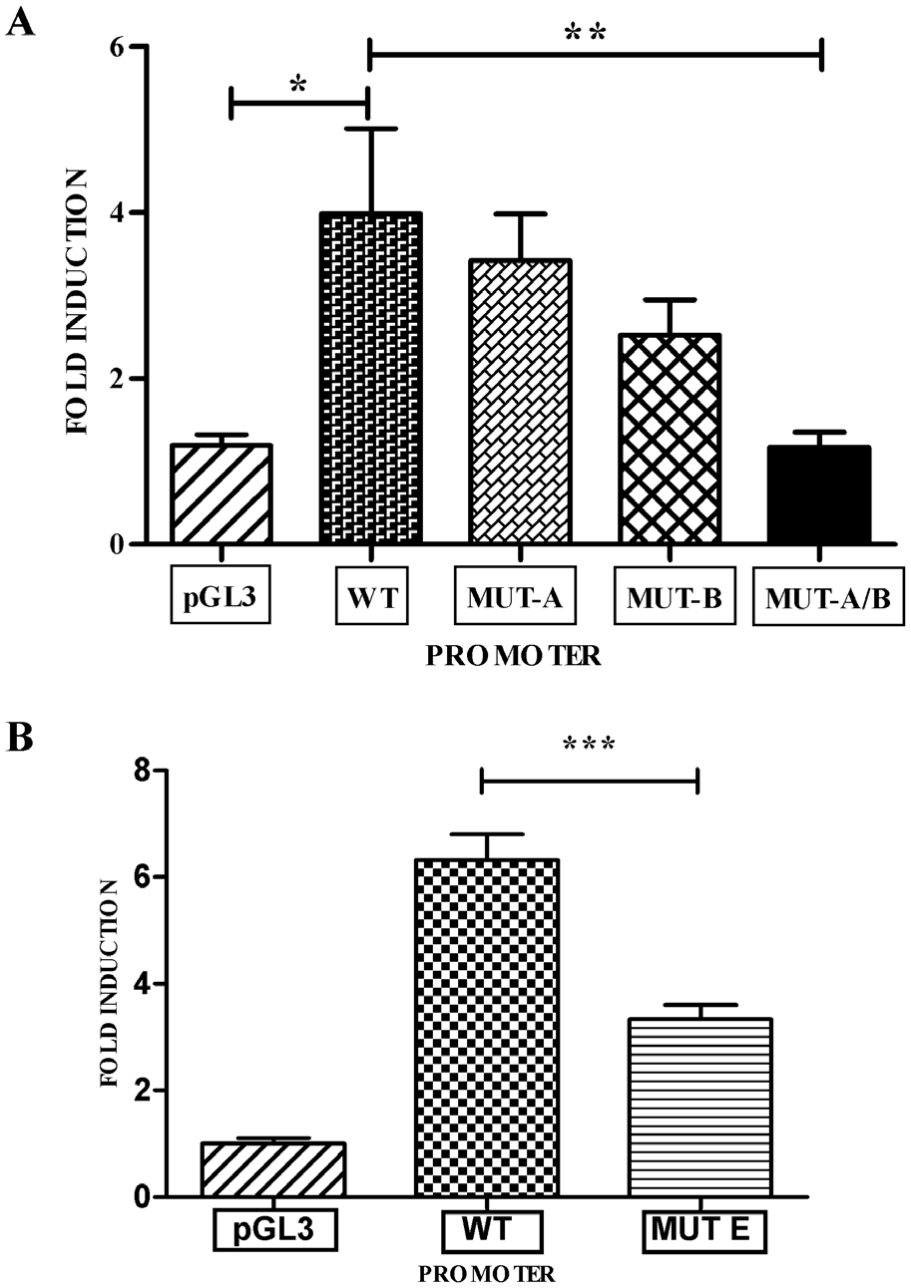
GLI Activators Induce Transcription at the Human *TGF-β1* Promoter. The human *TGF-β1* promoter region was placed into a pGL3-basic luciferase vector. (A) The three GLI binding sites within this region (Sites A, B, and C) were mutated separately or together (WT  =  wild-type *TGF-β1* promoter phTG5, MUT-A  =  site A mutation, MUT-B  =  sites B/C mutation, MUT-A/B  =  sites A/B/C mutation, pGL3 =  control vector). These *TGF-β1* promoter:luciferase constructs were co-transfected into 293T cells with the constitutive GLI activator (GLI2dN) and normalized to the negative control (pcDNA3). The GLI activator induced a four-fold increase of luciferase activity in phTG5 over the control. Mutation of both GLI binding sites (phTG5AB) abrogated this induction (n = 10, *p = 0.0011, ** p = 0.0033). (B) The putative GLI binding Site E was mutated in the reporter plasmid phTG1 (WT  =  wild-type TGF-1 promoter phTG1, MUT-E  =  site E mutation, pGL3 =  control vector). The GLI activator induced a six-fold increase of luciferase activity in phTG1 over the control. Mutation of GLI binding Site E decreased this induction nearly two-fold (n = 12, *** p = 0.0025).

Following co-transfection of GLI2ΔN and phTG5 into 293T cells, we observed an approximately 4-fold increase of luciferase expression over the controls (Figure 2). As the promoter region of phTG5 contains three of the six putative GLI binding sites (A, B, & C) we used site-directed mutagenesis to mutate these putative GLI binding sites individually or in combination (denoted phTG5A, phTG5B, and phTG5AB respectively). Although mutation of a single site only modestly reduced luciferase expression, mutation of both sites reduced reporter gene expression down to control levels. These findings indicate that GLI2 can activate transcription at the human *TGF-β1* promoter and that binding to at least one of these sites is required for this to occur. Mutation at Site E, which lies further upstream of the transcription start site, was done to assess its role in GLI2-induced TGF-β1 expression. We used a luciferase reporter construct phTG1 containing −1362 to +11 bases near the transcription start site of the human TGF-β1 promoter [18]. Following mutation of the possible GLI-binding site, the luciferase expression was decreased approximately two-fold following cotransfection with the constitutive GLI2 activator. Further analysis will be needed to examine whether putative GLI binding sites other than sites A, B, and E are also functional.

**Figure 3:**
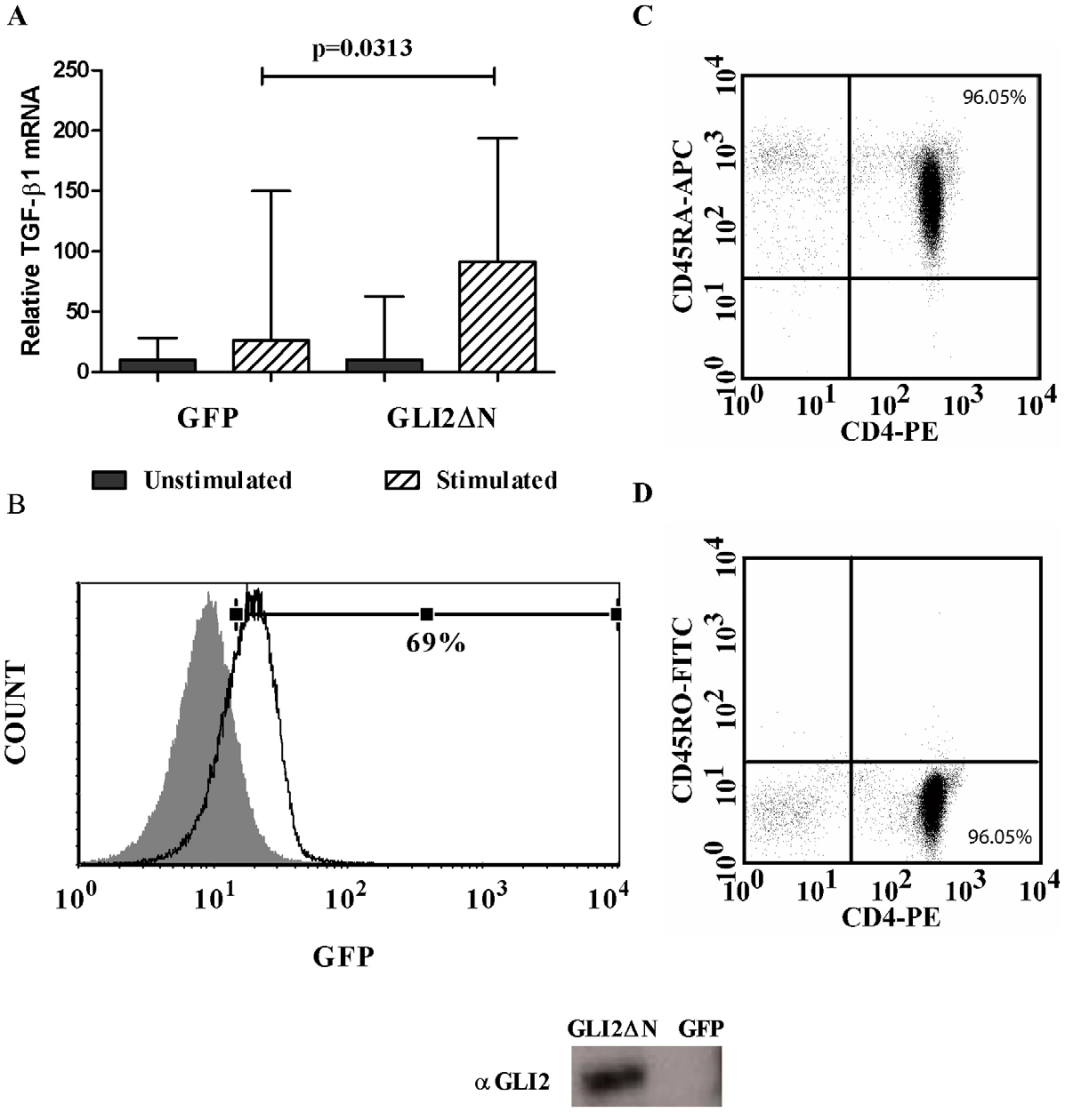
Lentiviral Infection of Naïve CD4^+^ T cells with GLI2 Activator Increases *TGF-β1* transcription. Naïve human CD4^+^ T cells were isolated by negative selection and infected with a lentivirus containing a GLI2ΔN activator construct or a GFP control. 72 hours post-infection, cellular RNA was collected to measure *TGF-β1* transcripts. (A) Relative *TGF-β1* mRNA was measured by RT-PCR and normalized to 18S rRNA. *TGF-β1* mRNA was significantly increased (n = 6, * p = 0.0313) in cells infected with GLI2ΔN as compared to cells infected with GFP control. (B) The infection efficiency was measured at 69% using a GFP expressing control lentivirus. (C & D) The phenotype of the naïve CD4^+^ T cells was greater than 95% of the cells at Day 0 were CD4^+^CD45RA^+^CD45RO^-^ naïve T cells. Expression of GLI2ΔN was confirmed by Western Blot (insert).

### Activated GLI2 Can Enhance *TGF-β1* Transcription in Naïve CD4^+^ T cells

In order to investigate the effect of the GLI transcription factors in primary cells, we infected primary naïve CD4^+^ T cells with lentiviral vectors containing the constitutive activator GLI2ΔN or a GFP control. Expression of GLI2ΔN was confirmed by Western blot (see insert in Figure 3). Naïve CD4^+^ T cells do not constitutively express high levels of TGF-β1. We measured the level of *TGF-β1* mRNA by RT-PCR before and after infection with the GLI constructs. As shown in Figure 3A, overexpression of the constitutively activated GLI2ΔN in naïve CD4^+^ T cells significantly increased *TGF-β1* mRNA expression (n = 6, p = 0.0313) following stimulation with αCD3/αCD28-coated beads. Upregulation of *GLI1* mRNA, a known GLI2-mediated gene, indicates that the GLI2ΔN protein is activating transcription in these lentivirally infected cells (data not shown). The infection efficiency was measured by infecting naïve CD4^+^ T cells with a control lentivirus expressing GFP (69% infection efficiency, Figure 3B). The phenotype of the naïve cells was CD4^+^CD45RA^+^CD45RO^-^ (Figures 3C & 3D).

**Figure 4:**
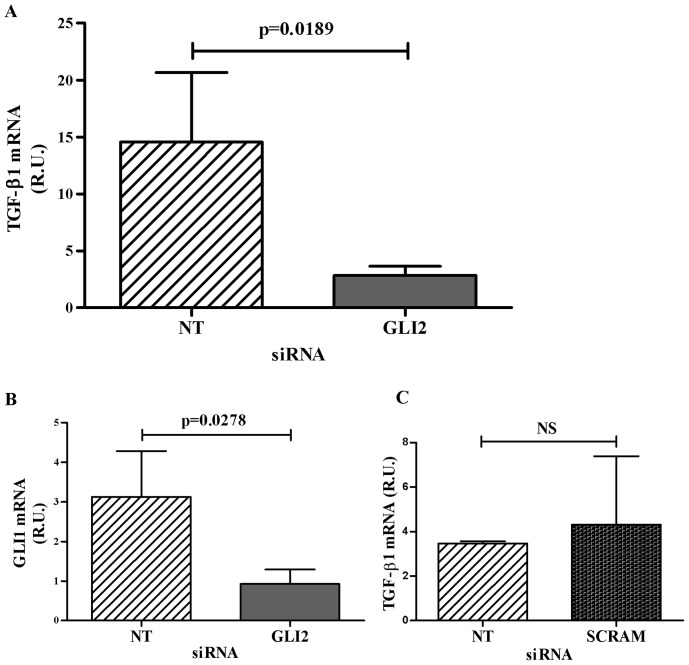
GLI2 Knockdown in CD4^+^CD25^+^FoxP3^+^ Treg Decreases Levels of *TGF-β1* mRNA. CD4^+^CD25^+^FoxP3^+^ Treg were enriched from PBMC and then incubated for 72 hours with non-targeting (NT) or *GLI2* siRNA. The cells were then stimulated (1 αCD3/αCD28 bead: 1 cell) for an additional 24 hours. RT-PCR was used to measure *TGF-β1* mRNA levels. Relative *TGF-β1* or *GLI1* mRNA was normalized to 18S rRNA. (A) *GLI2* knockdown in Treg significantly decreased *TGF-β1* transcription (n = 7, * p = 0.0189). (B) *GLI1* mRNA was also measured as a control GLI2-regulated gene; showing that knockdown of *GLI2* in Treg also diminished *GLI1* mRNA (n = 5, ** p = 0.0278). (C) The sequence of the *GLI2* siRNA was scrambled (SCRAM) as an additional *GLI2* siRNA negative control and was not significantly different from the non-targeting control.

### GLI2 Regulates *TGF-β1* Transcription in Primary Human CD4^+^ CD25^hi^ Treg

Although overexpression of transgenic GLI proteins can modulate *TGF-β1* transcription in naïve CD4^+^ T cells, we wanted to assess the physiological role of GLI2 activation in primary cells that express high levels of TGF-β1. Previous studies have used siRNA to knockdown specific genes in primary Treg [31], [32]. Knockdown of *GLI2* was done using commercially available Dharmacon ACCELL siRNA to assess its function in TGF-β1 transcription in primary human Treg. *GLI2* siRNA knockdown in primary human CD4^+^CD25^hi^ Treg stimulated with αCD3/αCD28 coated beads decreased the expression of *TGF-β1* mRNA by approximately 5-fold (Figure 4A, p = 0.0189). To assess the knockdown of GLI2 activity, *GLI1* mRNA expression was measured by RT-PCR. *GLI1* gene transcription is a known downstream target of the activated GLI2 transcription factor. The decreased expression of *GLI1* mRNA supports the siRNA-mediated knockdown of *GLI2* (Figure 4B, p = 0.0278). A separate independent negative control using a scrambled *GLI2* siRNA sequence (5'- AAGGUAUGCGCUUAAUUU-3') was used to address off-target effects (Figure 4C). These results indicate that GLI2 is important in regulating *TGF-β1* transcription in primary human Treg. The sorted Treg were CD3^+^CD8^-^CD4^+^CD25^hi^FoxP3^+^ T cells. The purity of the Treg was more than 85% (Figure 4D).

**Figure 5:**
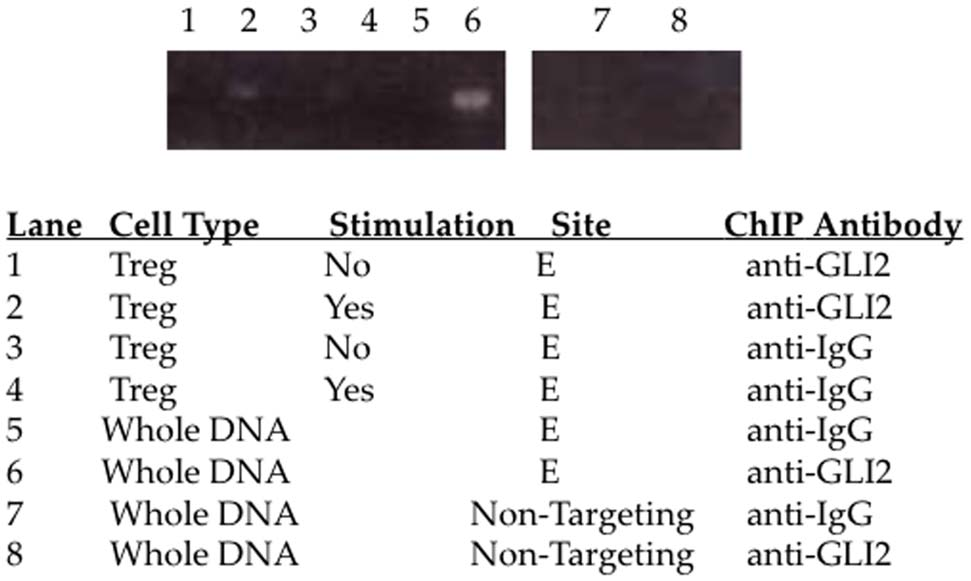
GLI2 binds to the human *TGF-β1* promoter. Chromatin immunoprecipitation (ChIP) was done to assess the binding of GLI2 to the human *TGF-β1* promoter. Primary human regulatory CD4^+^CD25^hi^ T cells were negatively isolated and stimulated with αCD3/αCD28-coated beads overnight. PCR was done to assess GLI2 binding. Whole DNA was used as a positive control for the PCR. GLI2 bound to the *TGF-β1* promoter at ‘Site E’ in the human *TGF-β1* promoter. GLI2 did not bind to a non-targeting region in the human *IL-10* promoter (Lane 8).

### GLI2 Binds to the Human *TGF-β1* Promoter

Chromatin immunoprecipitation (ChIP) was done to assess the binding of GLI2 to the human *TGF-β1* promoter. Primary human regulatory CD4^+^CD25^hi^ T cells were negatively isolated and stimulated with αCD3/αCD28-coated beads overnight. The stimulated Treg were incubated with agarose beads coated with αGLI2 or αIgG control antibodies. As shown in Figure 5, GLI2 bound to the *TGF-β1* promoter. GLI2 binding was shown to be at ‘Site E’ (Figure 5) in the human *TGF-β1* promoter. However GLI2 did not bind to a non-targeting region in the human *IL-10* promoter (Lane 8).

**Figure 6:**
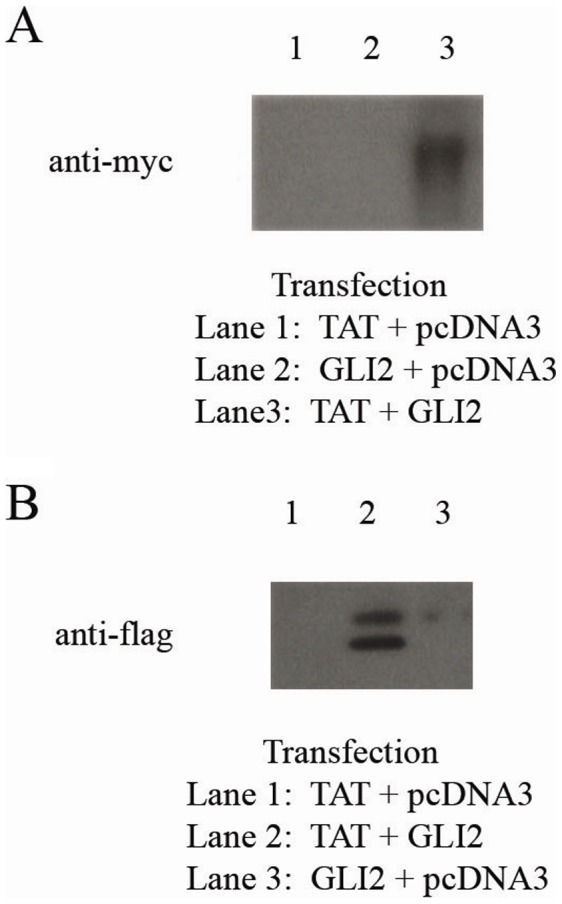
HIV-1 Tat Binds to Human GLI2. Co-immunoprecipitation was used to test the binding of HIV-1 Tat to human GLI2. Dual transfections were done in 293T cells (plasmid combinations shown on right) to express the Tat-FLAG and GLI2-6xMYC proteins (pcDNA3 = empty control vector). (**A**) Beads were coated with anti-FLAG antibody and incubated with cell lysates from transfections #1–3 shown on right-hand side. Following washing and elution steps, the bound protein was run on a SDS-PAGE gel followed by Western Blotting to assess GLI2 binding. Anti-myc antibody was used to confirm binding of GLI2 to Tat when Tat was bound to the bead. (**B**) Beads were coated with anti-myc antibody and incubated with cell lysates from transfections #1–3 shown on right-hand side. Following washing and elution steps, the bound protein was run on a SDS-PAGE gel and Western Blotting was done to look for HIV-1 Tat binding. Bands developed at approximately 27 kDa. Anti-flag antibody was used to confirm binding of Tat to GLI2 when GLI2 was bound to the bead.

### HIV-1 Tat Binds to GLI2

Increased plasma and cerebral spinal fluid (CSF) levels of immunosuppressive cytokines like TGF-β1 are found during HIV-1 disease progression[8]–[10] leading to an increase in iTreg [11], [12]. The HIV-1 transactivator and viral protein Tat has been implicated as the inducer of TGF-β1 in both *in vitro* as well as *in vivo* models [13]–[17]. Although Tat has been shown to increase TGF-β1, the underlying transcriptional mechanism has not yet been clearly defined.

Since we found that GLI2 regulates TGF-β1 at the transcriptional level, we tested whether HIV-1 Tat binds to human GLI2 and can thereby increase or stabilize TGF-β1 transcription using co-immunopreciptation (co-IP) to assess the binding of human GLI2 to HIV-1 Tat. Dual transfections were done in 293T cells with three different sets of plasmids: (GLI2-6xMYC + HIV-1 Tat-FLAG), (GLI2-6xMYC + PCDNA3 control), (HIV-1 Tat-FLAG + PCDNA3 control). Forty-eight hours post-transfection, the cells were lysed and the released proteins were collected for co-IP (Pierce Co-IP Kit from Thermo Scientific). The agarose resin was coated with one of three antibodies: Sigma F1804 anti-FLAG M2 antibody (to bind Tat to bead), CST 71D10 anti-MYC antibody (to bind GLI2 to bead), or an isotype control antibody. Following co-IP, the elutions were run on an SDS-PAGE gel followed by Western Blotting. As shown in Figure 6, HIV-1 Tat bound specifically to GLI2 which was bound to a bead. The reverse reaction also showed specificity, indicating that these two proteins can indeed interact. The binding of HIV-1 Tat to GLI2 suggests a potential mechanism for TGF-β1 induction in HIV-1 infection. Further experiments will need to be done to test the functional synergy of HIV-1 Tat and GLI2 to affect transcription at the TGF-β1 promoter.

## Discussion

Our results show that the transcription factor GLI2 plays a previously unknown, but important role in the complex transcriptional regulation of *TGF-β1.* Through *in silico* screening analysis we revealed six potential GLI-binding sites in the human TGF-β1 promoter, five of which were previously unknown. Using mutational analysis of the human *TGF-β1* promoter, we found that GLI2 enhances *TGF-β1* transcription in at least three of the five putative GLI-binding sites (Sites A, B, & E). In addition our results show that infection of primary naïve CD4^+^ T cells (low TGF-β1 expressing cells) with a constitutively active GLI2 lentivirus enhances *TGF-β1* transcription. Furthermore, siRNA knockdown of *GLI2* in primary human CD4^+^CD25^hi^FoxP3^+^ Treg (high TGF-β1 expressing cells) significantly decreased *TGF-β1* transcription. We also show by ChIP that GLI2 directly binds to “Site E” in the *TGF-β1* promoter. This newly identified mechanism GLI2-mediated TGF-β1 regulation may have implications in diseases such as cancer and AIDS, where high levels of TGF-β1 contribute to pathogenesis.

Although the role of GLI-mediated regulation of *TGF-β1* transcription has not previously been shown, there is indirect evidence for this mechanism. Eichenmuller *et al.* discovered that *Ptch1* knockout mice, which have constitutive activation of the SHH pathway, have more than 400-fold increased levels of *TGF-β1* mRNA transcripts [28]. Although the investigators found that the putative GLI binding site in the second promoter was not responsive to GLI1, they did not investigate the role of GLI2 in regulating the five putative GLI binding sites that we discovered in the first TGF-β1 promoter.

Additionally, several reports emphasize the close relationship between the TGF-β1 and SHH/GLI families [33], [34]. Liu *et al.* reported the interaction between the SHH pathway molecule GLI3 and the SMAD proteins (TGF-β1 pathway transcription factors) [35]. Additionally Dennler *et al.* reported that GLI2 and GLI1 could be induced through a SMAD-mediated mechanism [36]. The TGF-β superfamily genes *BMP-2, 4*, & *7* all have GLI-responsive elements in their promoters[37]–[39]. Lin *et al.* found a SHH-responsive element in the 5′-upstream promoter region of *TGF-β2* [40]. TGF-β1 and its effector molecule SMAD3 have been shown to upregulate *GLI2* and *GLI1* [36], [41]. Despite the extensive cross-regulatory network of TGF-β and SHH, the regulation of *TGF-β1* by SHH family members, such as the GLI proteins, has not been previously reported.

Several types of tumors (basal cell carcinomas, gliomas, medullablastomas, and prostate cancer) express abnormally high levels of GLI proteins or increased SHH pathway activation, which may be targets for therapy [42]. Although aberrant activation of SHH/GLI signaling leads to elevated expression of immunosuppressive factors like TGF-β1[43]–[49], a clear mechanism of cancer-induced TGF-β1 induction is still lacking. One possible mechanism is through activation of GLI2 by the ERK and AKT pathways in tumor cells. The ERK and AKT pathways are hyperactivated in several tumors and are critical for GLI-regulation in both tumor cells [50], [51] as well as primary mammalian cells [52], [53]. Our data indicate that the ERK and AKT pathways are also critical for GLI activation in CD4^+^ T cells (data not shown). The hyperactivation of the ERK and AKT pathways in tumor cells may provide an environment for GLI2-mediated transcription of the immunosuppressive TGF-β1.

We also show that HIV-1 Tat binds to GLI2. The interaction between HIV-1 Tat and GLI2 may be a contributing factor to the elevated levels of TGF-β1 in HIV infection and contributes to immune dysregulation, a key characteristic of chronic HIV-1 disease. Increased plasma and cerebrospinal fluid (CSF) levels of immunosuppressive cytokines like TGF-β1 are found during HIV-1 disease progression[8]–[10] leading to an increase in iTreg [11], [12]. This increase of immunoregulatory CD4^+^CD25^+^FoxP3^+^ T-cells (Treg) during chronic HIV infection disables the immune system’s control over viral replication as well as opportunistic infections [12]. The HIV-1 transactivator Tat has been implicated as the inducer of TGF-β1 in both *in vitro* as well as *in vivo* models [13]–[17], although the underlying transcriptional mechanism has not been clearly defined.

Our current findings support those of Browning *et al.*, who found that human GLI2 bound to HIV-1 Tat [54]. The role of the GLI proteins in retroviral replication have previously been reported by several groups[54]–[57]. Specifically, GLI-2 has been previously shown to interact with retroviral transactivators to increase viral transcription[54]–[57]. We suggest that the reported induction of TGF-β1 transcription by HIV-1 Tat[13]–[17] may be in part mediated by the binding of Tat to GLI2 at the *TGF-β1* promoter. Although further studies will need to be done to validate this mechanism, the novel role of GLI2 in *TGF-β1* transcription may provide an interesting link between HIV-1 replication and TGF-β1 induction.

The role of TGF-β1 in pathogenesis of cancer, AIDS, and several other diseases warrants interest in the regulation of this immunoregulatory cytokine. The regulatory role of GLI2 in *TGF-β1* transcription extends our understanding of how the SHH and TGF pathways interact in mammalian biology. How GLI2 is regulated by the ERK and AKT pathways and by HIV-1 Tat may lead to insight into TGF-β1 induction during cancer progression and HIV infection.
